# Effects of ecological environment and host genotype on the phyllosphere bacterial communities of cigar tobacco (*Nicotiana tabacum* L.)

**DOI:** 10.1002/ece3.7861

**Published:** 2021-07-21

**Authors:** Lei Xing, Jianlei Yang, Yuhong Jia, Xi Hu, Yi Liu, Heng Xu, Huaqun Yin, Juan Li, Zhenxie Yi

**Affiliations:** ^1^ College of Agronomy Hunan Agricultural University Changsha China; ^2^ Greatwall Cigar Factory China Tobacco Sichuan Industrial Co., Ltd Shifang China; ^3^ School of Minerals Processing and Bioengineering Central South University Changsha China

**Keywords:** bacterial communities, cigar tobacco, environmental conditions, genotype, phyllosphere, species diversity

## Abstract

Microorganisms of plant phyllosphere play an important role in plant health and productivity and are influenced by abiotic and biotic factors. In this study, we investigated the phyllosphere bacterial communities of three cigar tobacco varieties cultivated in Guangcun (GC) and Wuzhishan (WZS), Hainan, China. Metagenomic DNA was extracted from tobacco leaf samples and sequenced by 16S rDNA amplicon sequencing. Our results showed that bacterial communities of cigar tobacco phyllosphere in GC exhibited remarkably higher alpha diversity than that in WZS. There was slight effect of tobacco genotype variations on the alpha diversity in both cultivation sites, and beta diversity and structure of bacterial community were not influenced significantly by the cultivation sites and tobacco varieties. Statistical analyses of species diversity unraveled that the dominant species in bacterial communities of cigar tobacco phyllosphere among all these samples were phylogenetically affiliated to *Proteobacteria* and *Cyanobacteria*. At the genus level, the most abundant microorganism was *Limnobacter*, followed by *Brevundimonas*, unidentified_*Cyanobacteria*, and *Pseudomonas*. Additionally, environmental conditions except for humidity were negatively correlated with the relative abundance of bacterial genera. Further analyses revealed that influence of site‐specific factors on tobacco bacterial community was relatively higher than genotype‐specific factors. In short, this study may contribute to the knowledge base of practical applications of bacterial inoculants for tobacco leaf production.

## Graphical abstract

This study provided a theoretical support when applying the phyllosphere bacterial communities to promote tobacco growth, prevent disease, and regulate the tobacco aging process artificially.

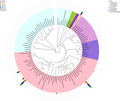

## 1 INTRODUCTION

Plant phyllosphere is defined as the aerial part of plants and dominated by leaves (Vorholt, 2012), and the leaf surface is considered as one of the largest habitats for microorganisms such as bacteria, yeasts, archaea, and fungi on earth (Legein et al., 2020; Wellner et al., 2011). Of these microorganisms, bacteria are the most prevalent colonizers in phyllosphere (Martirosyan et al., 2016). It is recognized that bacteria in phyllosphere have beneficial effects on the plants, including nutrient recycling, production of plant growth hormones, pathogenic control, and bioremediation of harmful chemicals (Gu et al., 2010; Thapa et al., 2017). For instance, *Methylobacterium* can provide growth‐promoting substances such as vitamin B12, cytokinins, and auxins for the improvement of seed germination and root development to increase the yield of plants (Wellner et al., 2011). In recent decades, bacteria in plant phyllosphere have thus aroused great concern in the world (Liu et al., 2020).

Recently, numerous efforts have been attempted to reveal the diversity and composition of bacterial communities in plant phyllosphere (Chen et al., 2018). Previous studies have shown that bacterial community structure of phyllosphere was affected by both of the abiotic factors, such as environmental conditions (e.g., solar UV radiation, temperature fluctuations, and nutrient), geographic locations, and agronomic measures, and biotic factors, such as microbial interaction, leaf characteristics, and genotypes of host plant (Grube et al., 2011; Gu et al., 2010; Legein et al., 2020; Vorholt, 2012). Genotypes affect bacterial communities related to plant. Adams found that there were significant differences in microbial community structure among different genotypes of cotton. Genotypes, geographical location, and land‐use could affect the community of *Proteobacteria*; normally, we can see the significant different community composition among meadows and mown pastures (Wellner et al., 2011).

Tobacco (*Nicotiana tabacum* L.) is an important agricultural nonfood crop and the main raw material of tobacco commodities worldwide (Chen et al., 2020; Lisuma et al., 2020). It is widely cultivated in South China, such as Hainan and Sichuan (Tang et al., 2020; Yuan et al., 2016; Zhao et al., 2007). In several years, studies on phyllosphere bacteria of tobacco plants have been recorded. Chen et al. (2021) analyzed the effect of a broad‐spectrum fungicide on the bacterial communities of tobacco leaves using 16S rDNA amplicon sequencing, suggesting that the plant phyllosphere was dominated by *Proteobacteria* and *Alphaproteobacteria*, which were accounting for 33.80% of bacterial communities. Previous studies have demonstrated that bacterial composition and diversity in the plant phyllosphere varied with the environmental conditions and plant genotypes (Kim et al., 2012). In addition, phyllosphere bacteria are significantly affected by environmental conditions (Tang et al., 2020). To our current knowledge, most of the studies focused on chemical and biological effects on the bacterial communities in phyllosphere and/or rhizosphere (Perazzolli et al., 2020; Thapa et al., 2017; Vorholt, 2012), while little is known how environmental conditions and host genotypes modulate the bacterial community of tobacco leaves.

In this study, structures of bacterial community in phyllosphere of three tobacco varieties from two geographic locations were investigated using 16S rDNA gene sequencing. The alpha and beta diversity and composition of bacterial communities were characterized. Additionally, the relations between soil nutrients/climatic factors and bacterial abundance were evaluated. Studies on bacterial communities of tobacco phyllosphere would facilitate us to advance our understanding of bacterial variation in the tobacco phyllosphere and serve as a basis for promoting plant growth and protection in the future.

## 2 MATERIALS AND METHODS

### 2.1 Field experiment

The experimental fields for cigar tobacco cultivation were located in Guangcun (GC, 19°49′N, 109°28′E) and Wuzhishan (WZS, 18°88′N, 109°40′E), Hainan, China. It is well known that Cuba is rich in high‐quality cigar materials. Because of the similarities with Cuba in climate and natural conditions, China's Hainan is recognized to be suitable for the growth of cigar. According to the evaluation results of sensory quality, three cigar varieties with stable quality (Hanyan101, Haiyan201 and Haiyan209) were selected to be planted in Guangcun and Wuzhishan. In this study, fields with moderate yield, medium and even soil fertility, and no diseases and insect pests were chosen. The nitrogen application rate was 180 kg/hm^2^, and the nutrient ratio was N: P_2_O_5_: K_2_O = 1:1:3. Each treatment has three plots, each plot is 90 m^2^, and the row spacing is 40 cm × 100 cm. Other field management measures were carried out according to the local planting technology.

### 2.2 Sampling of leaf

Sampling of leaf was done at the harvest period of tobacco in April 2019. Healthy leaves from 10 different tobacco plants were collected from each plot. Fresh tobacco leaves were cut by sterile scissors, placed into the sterilized centrifuge tubes, and stored on the ice of cooling box. After arrival in the laboratory, leaf samples were frozen at −20°C and analyzed within one month. The tobacco varieties of Hanyan101, Haiyan201, and Haiyan209 were labeled as M.1, M.2, and M.3, respectively. Leaf samples were named as GC.M.1, GC.M.2, GC.M.3, WZS.M.1, WZS.M.2, and WZS.M.3, respectively.

### 2.3 Extraction of genome DNA and PCR amplification

Total genome DNA from leaf samples was extracted using CTAB method (Huang et al., 2010). DNA concentration and purity were monitored by 1% agarose gel electrophoresis. V5–V7 hypervariable regions of 16S rRNA gene were amplified using specific pair of primers 799F (AACMGGATTAGATACCCKG) and 1193R (ACGTCATCCCCACCTTCC). PCRs were performed with 15 µl Phusion^®^ High‐Fidelity PCR Master Mix (New England Biolabs), 10 ng template DNA, and 2 µM forward and reverse primers. Negative control without DNA template was performed for each primer pair. Thermal cycling comprised initial denaturation for 1 min at 98°C, followed by 30 denaturation cycles for 10 s at 98°C, annealing for 30 s at 50°C, and elongation for 30 s at 72°C, with a final step of 72°C for 5 min. In order to evaluate the amplification result, amplified DNA sequences were measured via 2% agarose gel electrophoresis. Then, the PCR mixture was purified by Qiagen Gel Extraction Kit (Qiagen, Germany) according to the instructions.

### 2.4 Library construction and sequencing

Sequencing libraries were constructed with TruSeq^®^ DNA PCR‐Free Sample Preparation Kit (Illumina, USA) according to the manufacturer's recommendations. The library quality was evaluated by the Qubit@ 2.0 Fluorometer (Thermo Scientific) and Agilent Bioanalyzer 2100 system. Finally, the library was sequenced by an Illumina NovaSeq platform. Primers and barcodes were trimmed by the split library script available in QIIME. Reads shorter than 150 bases were discarded.

### 2.5 Soil nutrients and climatic conditions

Soil samples were randomly collected from the rhizosphere of different plants in the depth of 20 cm using a soil auger at each plot. Two replicates were carried out for each sampling site. The soil nutrients of the samples were determined by the standard methods: (a) organic matter (OM), NY/T1121.6‐2006, (b) total nitrogen (TN) and hydrolysable nitrogen (HN), LY/T1228‐2015, (c) total phosphorus (TP) and available phosphorus (AP), NY/T1121.7‐2014, and (d) total potassium (TK) and available potassium (AK), NY/T889‐2004. Air relative humidity was recorded by a hygroscope. Air temperature was measured by a thermometer. The total rainfall (R) and photosynthetically active radiation (PAR) were obtained from Hainan meteorological observatory.

### 2.6 Statistical analyses

Sequence analyses were carried out by Uparse software (Uparse v7.0.1001, http://drive5.com/uparse/; Edgar, 2013). Sequences with ≥97% similarity were assigned to the same operational taxonomic unit (OTU) for taxonomic assignments. Representative sequence of each OTU was used for further annotation. Venn diagrams were created for OUTs. The most influential taxa between groups were identified by *t* test with R software (Version 2.15.3). Phylogenetic analysis of the top 100 genera was conducted by R software with the maximum likelihood method.

Alpha diversity including Observed species, Chao1, Simpson, Shannon, and ACE was estimated for the samples using QIIME (Version 1.7.0) and displayed with R software. The significant differences of alpha diversity were further evaluated by two‐way analysis of variance (ANOVA) and Tukey multiple comparison using SPSS 19.0 software (SPSS Inc., Chicago, USA). For beta diversity analysis, Principal coordinate analysis (PCoA) plots were generated by WGCNA, stats, and ggplot2 package in R software. The significant levels of beta diversity between groups were further evaluated by Tukey's test. The community structure differences between groups were analyzed by ANOSIM based on Bray–Curtis distance (R software, vegan package). Statistical significance was set at *p* < .05.

The correlations between environmental factors and the relative abundances of keystone species were determined by Spearman's correlation analysis. The analysis was carried out with psych package and displayed with pheatmap package in R software.

## 3 RESULTS

### 3.1 Diversity of bacterial communities

**FIGURE 1:**
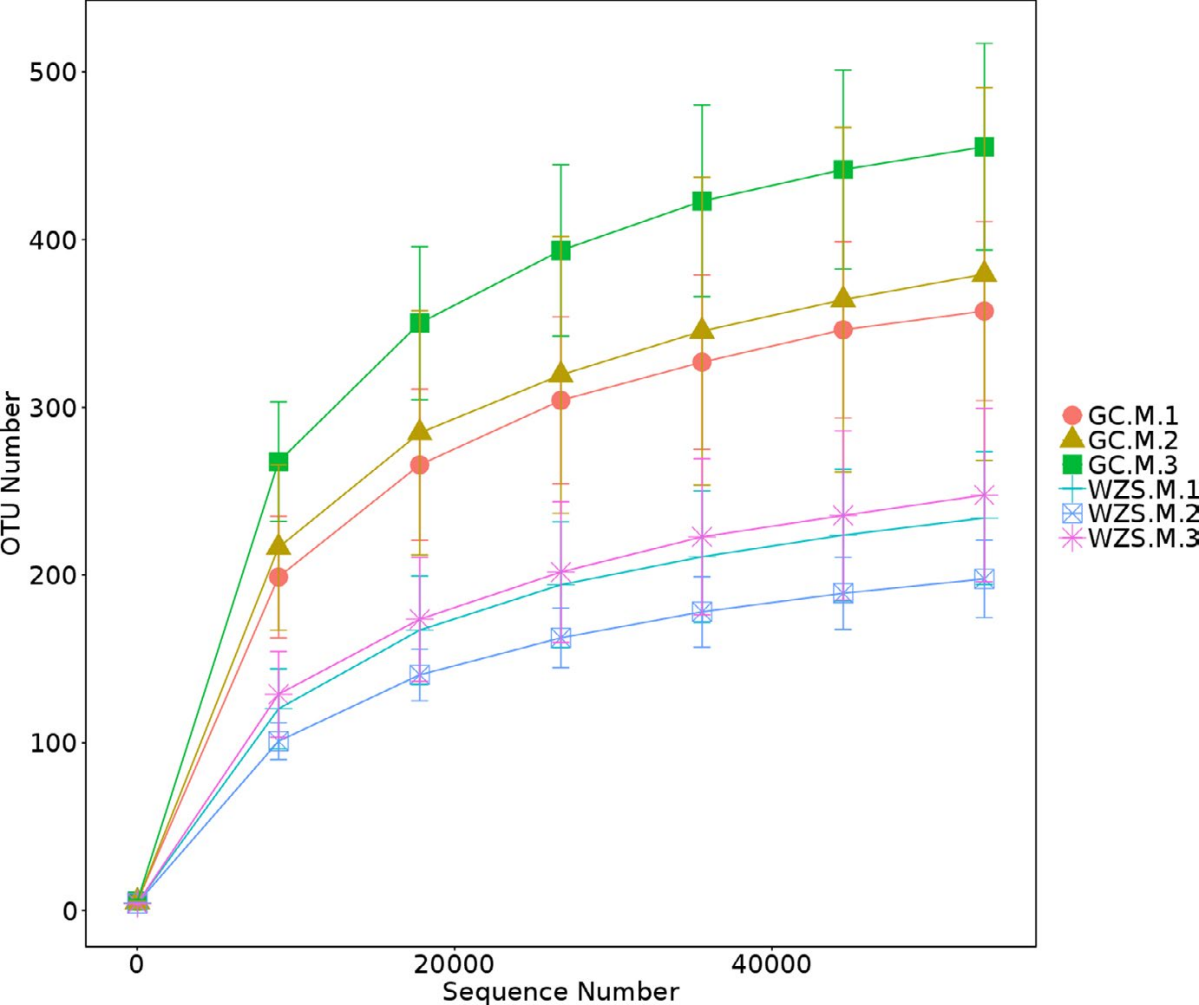
Rarefaction curves of OTUs across different samples

After quality filtering and removal of nonbacterial sequence reads, a total of 1,171,116 high‐quality (HQ) sequences were generated, with a range of 55,833 to 69,939 reads per sample. As shown in Figure 1, rarefaction curve of each sample tended to approach the plateau phase, when the amount of sequencing data reached approximately 40,000. This indicated that the number of sequences in these samples was sufficient to represent the bacterial composition (Chen et al., 2020).

**FIGURE 2:**
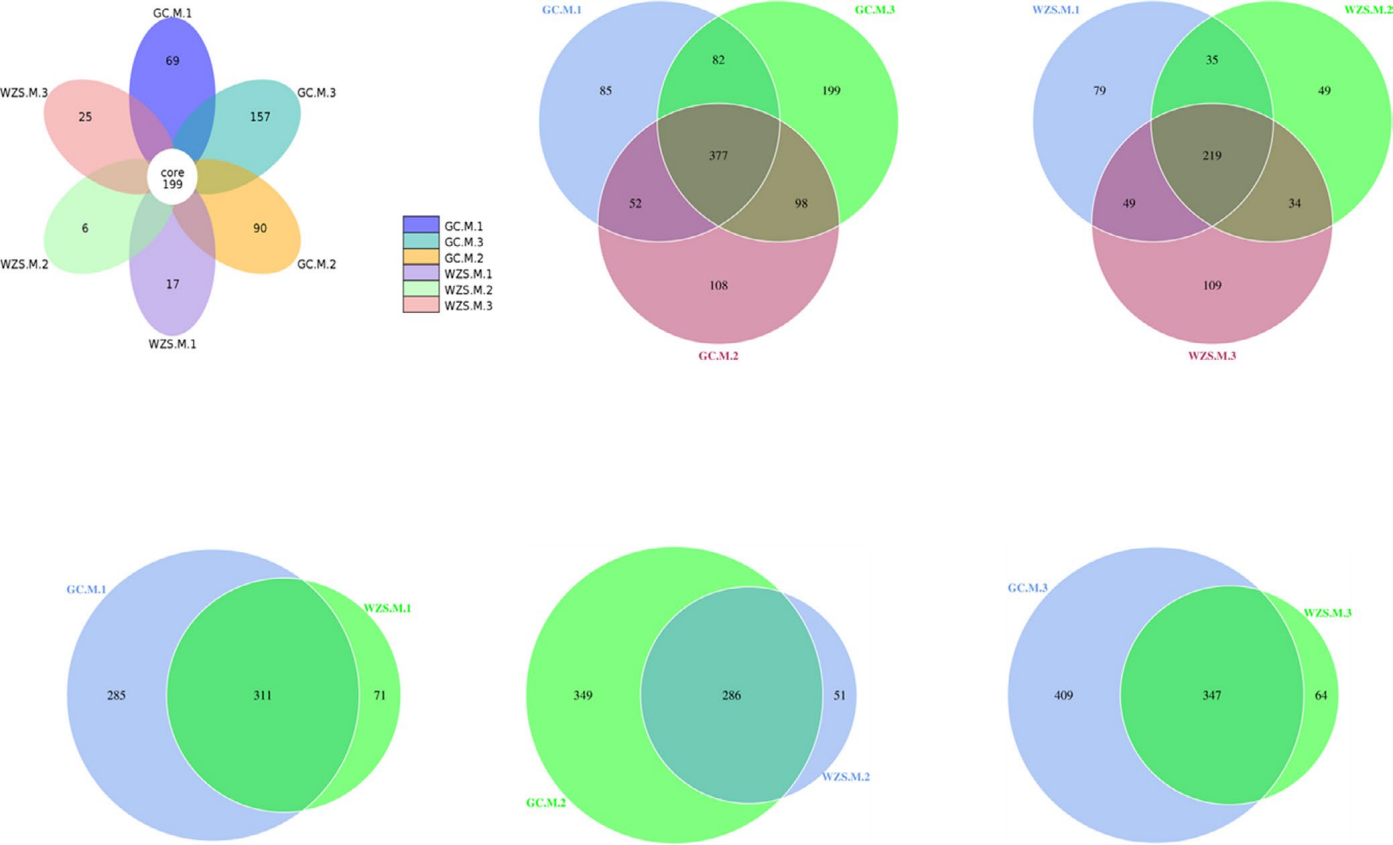
Venn diagrams of bacterial OTUs of all the samples. Numbers in the core sections denote shared OTUs of the samples. Numbers in the overlapping regions of the adjacent samples demonstrate shared OTUs. Numbers in the nonoverlapping regions of the samples mean unique OTUs

For taxonomic assignments, all HQ sequences were then classified by OUTs. Differences in community composition among different varieties from two distinct geographic regions (i.e., GC and WZS) were indicated by Venn diagrams (Figure 2). A total of 1,083 OTUs were identified among all environmental samples, in which 199 OTUs were shared. As for samples in GC, up to 1,001 OTUs were identified, of which 377 were assigned into the shared OTUs. In contrast, only 574 OTUs were detected in the samples from WZS, and 38.15% of them were shared OTUs. Furthermore, the same tobacco variety planted in different ecological regions showed a significant difference in terms of bacterial OTUs. The findings showed that bacterial diversity in phyllosphere of each tobacco variety from GC was relatively higher than that from WZS. More specially, samples from tobacco variety M.3 contained more total OUTs, unique, and shared OTUs compared with the others.

The diversity indices such as Observed species, ACE, and Chao1 indicate the abundance of bacterial communities. The Shannon and Simpson values reflect the diversity of the bacterial communities. Generally speaking, the larger the indices, the greater the abundance or diversity of the bacterial species. Further analysis revealed that all alpha diversity indices for bacterial communities in GC were significantly higher than that in WZS (*p* < .05) (Table 1). In addition, M.3 variety showed the highest diversity than M.1 and M.2 in both geographic locations. However, there was no significant difference in alpha diversity among different cultivars, regardless of the locations (*p* > .05; Table 2).

**TABLE 1:** Alpha diversity indices of bacterial communities

Groups	Observed species	Shannon	Simpson	Chao1	ACE
GC.M.1	357.33 ± 53.28	3.43 ± 0.26	0.83 ± 0.015	447.48 ± 69.96	411.78 ± 56.09
GC.M.2	379.33 ± 111.07	3.24 ± 0.34	0.81 ± 0.025	573.09 ± 297.59	456.92 ± 175.77
GC.M.3	453.33 ± 61.57	3.64 ± 0.52	0.82 ± 0.60	546.11 ± 74.78	501.33 ± 66.48
WZS.M.1	234.00 ± 39.66	2.64 ± 0.14	0.76 ± 0.025	306.54 ± 42.59	282.36 ± 41.06
WZS.M.2	197.67 ± 23.10	2.51 ± 0.073	0.74 ± 0.0057	287.97 ± 17.93	249.29 ± 28.60
WZS.M.3	247.67 ± 51.75	2.69 ± 0.094	0.77 ± 0.015	361.51 ± 65.43	315.15 ± 66.65

**TABLE 2:** Results of *p* values about the influence of sampling sites and varieties on alpha diversity of bacterial communities

Source of variation	Observed species	Shannon	Simpson	Chao1	ACE
	*p*				
Sites	.0010^*^	.001^*^	.002^*^	.021^*^	.005^*^
Varieties	.334	.376	.460	.710	.564
Site and variety interactive effect	.635	.859	.901	.738	.809

Note

Significant differences are indicated with *p < .05.

**FIGURE 3:**
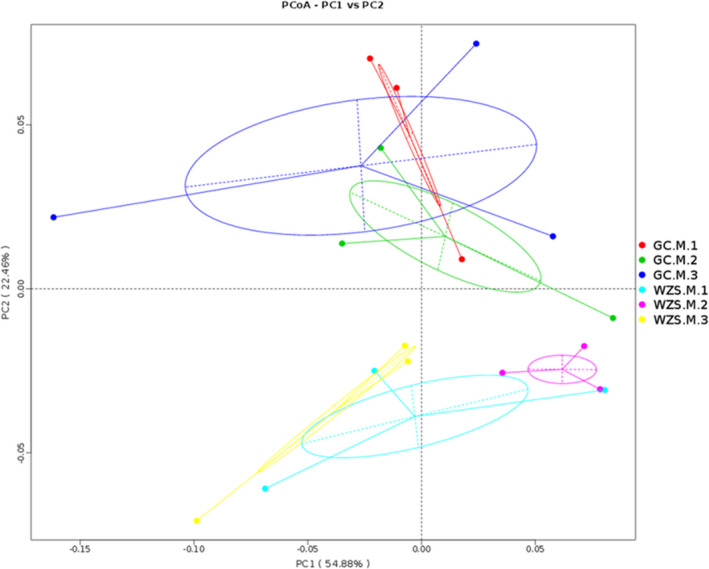
PCoA plot of the samples based on weighted Unifrac distance

Furthermore, PCoA plot based on weighted UniFrac distance matrix demonstrated the beta diversity for bacterial communities in tobacco phyllosphere. As depicted in Figure 3, the first and second principal components accounted for 54.88% and 22.46% of variance, respectively. The samples in the two ecological regions were obviously distinguished. The different varieties in GC and WZS exhibited the overlap to a certain extent.

### 3.2 Bacterial taxa in the tobacco phyllosphere

**FIGURE 4:**
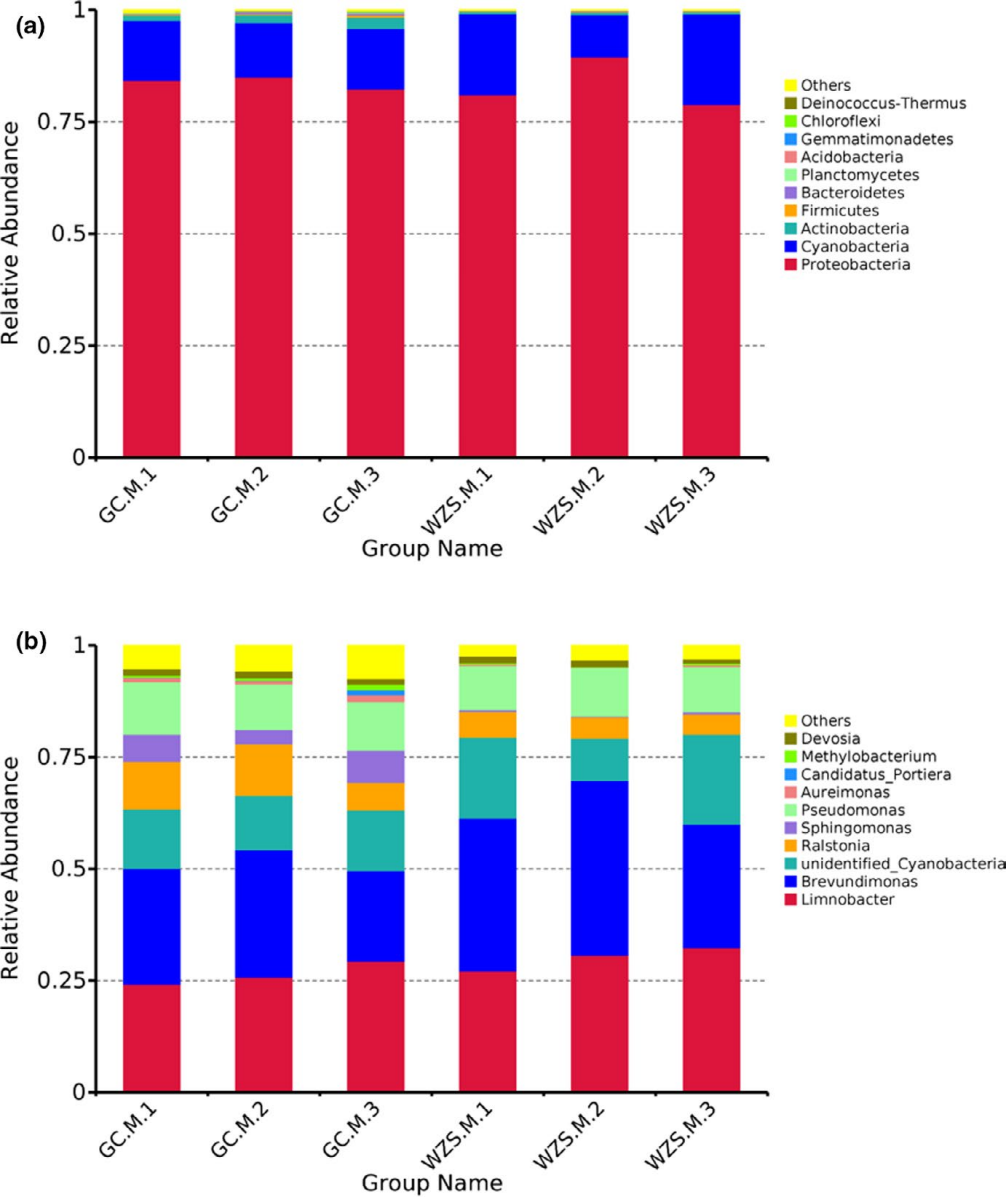
The relative abundance of bacterial species for the samples at phylum level (a) and genus level (b). The top ten abundant taxa were displayed

At the phylum level, the top ten bacterial species of all samples are shown in Figure 4a. The predominant bacterial phylum was *Proteobacteria*. The relative abundance of *Proteobacteria* for the three varieties in GC was similar (82.37%–85.00%), whereas the abundance of *Proteobacteria* in WZS samples had obvious differences, which ranged from 78.93% to 89.51%. The second most abundant bacterial species belonged to phylum *Cyanobacteria*. The relative abundance of *Cyanobacteria* in GC samples had no significant difference (12.17%–13.55%), while their relative abundance in WZS samples showed remarkable changes (9.44%–20.17%). M.2 cultivar revealed the relatively higher abundance of *Proteobacteria* and lower abundance of *Cyanobacteria* compared with the other varieties, regardless of the geographic locations. In contrast, M.3 cultivar exhibited the highest abundance of *Cyanobacteria* and lowest abundance of *Proteobacteria*. In addition, many other phyla such as comprised *Actinobacteria* (0.50%–2.5%), *Firmicutes* (0.11%–0.40%), and *Bacteroidetes* (0.057%–0.49%) were identified. These phyla in the tobacco samples from GC revealed relatively higher abundance than that in WZS.

Figure 4b described the relative abundance of the top ten bacterial genera in the samples, such as *Limnobacter*, *Brevundimonas*, unidentified_*Cyanobacteria*, *Ralstonia*, and *Sphingomonas*. The relative abundance of *Limnobacter* in GC samples varied from 24.29% to 29.44%, while *Limnobacter* exhibited slightly higher abundance (27.17%–32.46%) in WZS samples. The relative abundance of *Brevundimonas* in samples of GC and WZS corresponded to 20.21%–28.47% and 27.53%–39.05%, respectively. The relative abundance of *Limnobacter* in different tobacco varieties in order was M.3 > M.2 > M.1, while M.2 variety showed the highest relative abundance of *Brevundimonas*, followed by M.1 and M.3, regardless of the sampling sites.

Unidentified_*Cyanobacteria* remained constant composition (12.17%–13.53%) in GC, while its abundance varied from 9.44% to 20.16% in WZS according to different varieties. Unidentified_*Cyanobacteria* was most abundant in M.3 variety, followed by M.1 and M.2 varieties. The relative abundance of *Pseudomonas* in each sample was stable (9.90%–11.75%). *Ralstonia* and *Sphingomonas* represented higher abundance in the samples from GC (6.23%–11.60% and 3.18%–7.20%, respectively) than that in WZS (4.56%–5.71% and 0.16%–0.47%, respectively), whereas different varieties showed different relative abundance of *Ralstonia* and *Sphingomonas* in the same ecological region.

**FIGURE 5:**
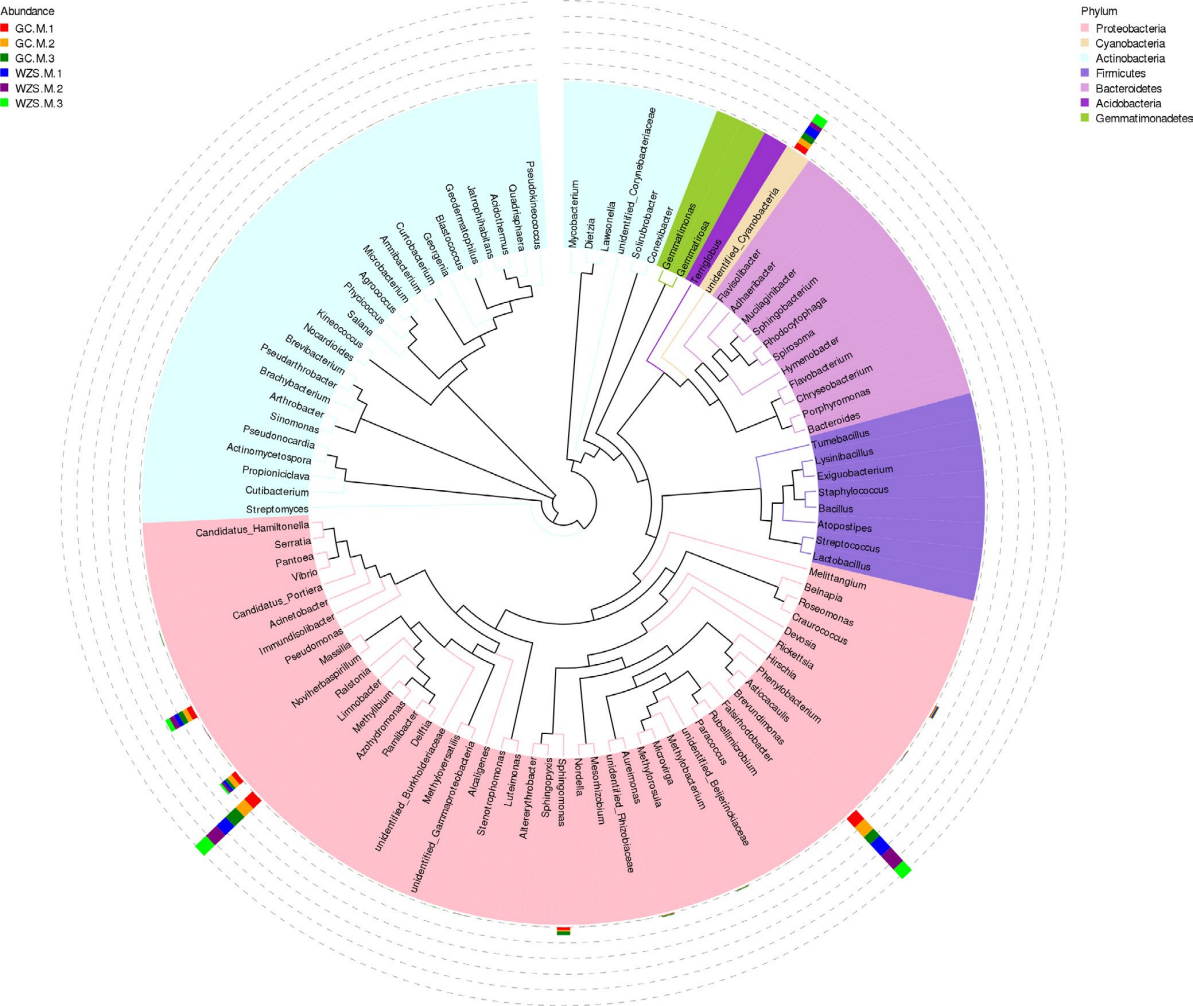
Maximum likelihood tree of the top 100 abundant bacteria genera for the six samples

A maximum likelihood tree based on the top 100 abundant bacterial genera (Figure 5) revealed that the most common bacterial genera were assigned to phylum *Proteobacteria*, followed by *Cyanobacteria*. Regarding the *Proteobacteria*, the dominant genera were *Limnobacter*, *Brevundimonas*, *Pseudomonas*, and *Ralstonia*. As for the *Cyanobacteria*, the dominant genus corresponded to unidentified_*Cyanobacteria*.

The taxa which showed significant differences of abundance between samples were also identified (Table S1). Relative abundance of four bacterial genera in M.2 variety displayed significant differences between the sampling sites (*p* < .05), while eight bacterial genera exhibited remarkable differences in relative abundance between the sampling sites for M.3 variety (*p* < .05). All these bacterial species such as *Melittangium*, *Blastococcus*, and *Roseomonas* presented significantly higher abundance in GC than that in WZS. Four bacterial genera differed significantly in relative abundance between M.2 and M.3 varieties in WZS (*p* < .05). The relative abundance of *Brevundimonas*, *Devosia*, and *Hirschia* was higher in M.2 compared with M.3, whereas *Agrococcus* showed higher abundance in M.3 than M.2.

### 3.3 Correlation between bacterial community structure and environmental variables

The climatic factors of these two geographic locations are described in Table 3. The mean air temperature (T) in GC and WZS was 23°C. The average relative humidity (H) was measured at 84% and 78% in GC and WZS, respectively. WZS revealed higher total rainfall (R) and photosynthetically active radiation (PAR) compared with GC. R and PAR for WZS were 132 mm and 338 µmol m^−2^ s^−1^, respectively, while GC exhibited the R and PAR at 89 mm and 309 µmol m^−2^ s^−1^, respectively.

**TABLE 3:** Climate factors for the tobacco cultivation

Ecological regions	T (°C)	H (%)	R (mm)	PAR (µmol m^−2^ s^−1^)
GC	23	84	89	306
WZS	23	78	132	338

**FIGURE 6:**
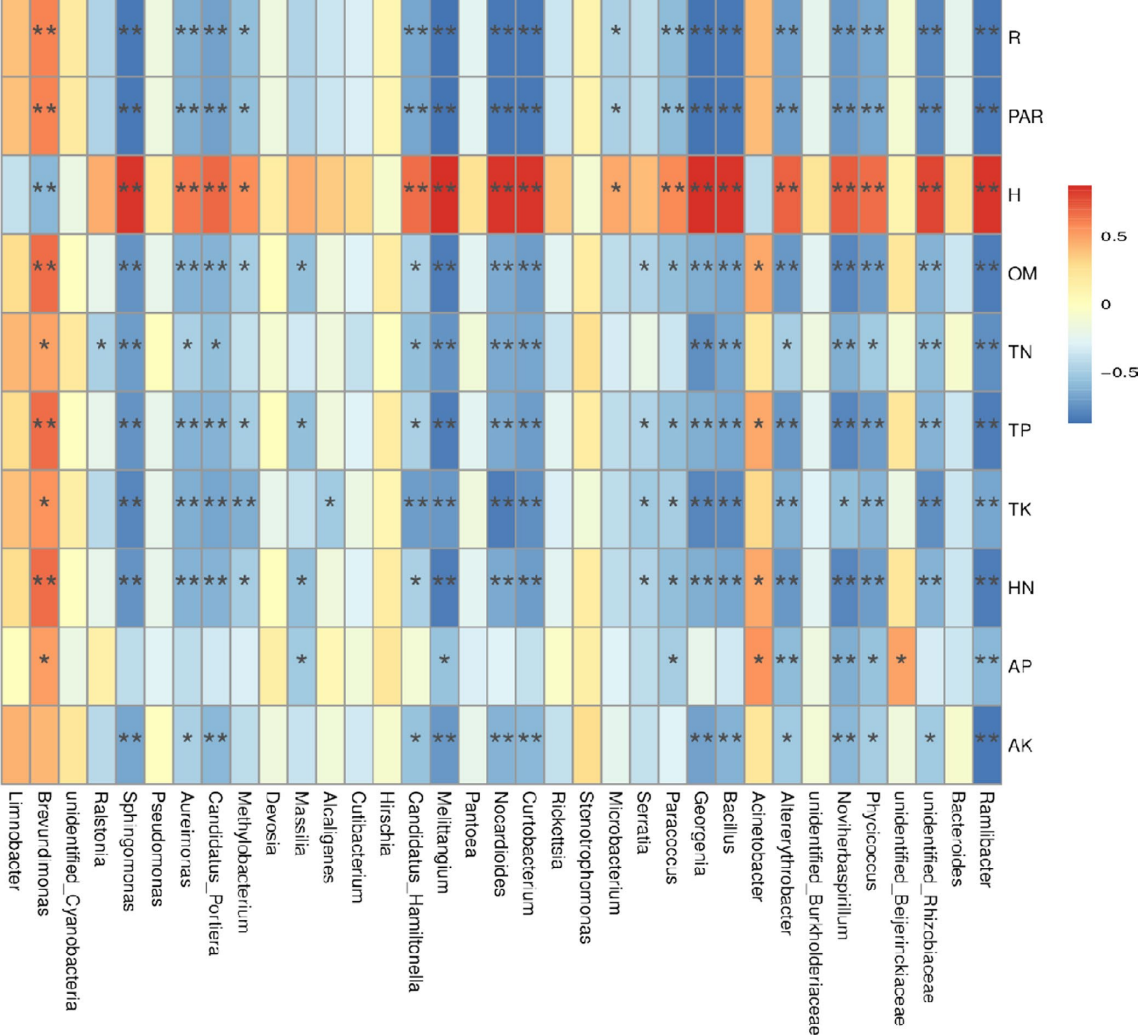
Spearman correlation analysis heat map between relative abundance of major genera and environmental factors. Temperature (T), total rainfall (R), relative humidity (H), photosynthetically active radiation (PAR), organic matter (OM), total nitrogen (TN), total phosphorus (TP), total potassium (TK), hydrolysable nitrogen (HN), available phosphorus (AP), available potassium (AK). * and ** indicate *p* < .05 and *p* < .01, respectively

Furthermore, soil macronutrient analysis revealed that WZS was more fertile than GC (Table 4). WZS exhibited the OM, TN, TP, HN, and AK at 23.42, 1.51, 1.32, 122.98, and 93.36 mg/kg, respectively, which were all approximately 2 times of that in GC. TK content in WZS (25.68 g/kg) was more than 20 times as much as TK content in GC (1.21 g/kg). AP content in WZS (35.46 mg/kg) was slightly higher than that in GC (32.74 mg/kg). The correlations between climatic/soil nutrient conditions and the relative abundance of dominant bacterial species were analyzed by Spearman correlation analysis (Figure 6). Results showed that the relative abundance of some bacterial genera such as *Sphingomonas*, *Aureimonas*, *Melittangium*, *Nocardioides*, and *Curtobacterium* was negatively related to R, PAR, OM, TN, TP, TK, HN, and AK, while they were positively correlated with H (*p* < .05). In contrast, the relative abundance of *Brevundimonas* was positively correlated with most of the environmental factors except H (*p* < .05).

**TABLE 4:** Soil nutrient conditions in two ecological regions

Ecological regions	OM (g/kg)	TN (g/kg)	TP (g/kg)	TK (g/kg)	HN (mg/kg)	AP (mg/kg)	AK (mg/kg)
GC	11.70	0.53	0.47	1.21	76.81	32.74	48.3
WZS	23.42	1.51	1.32	25.68	122.98	35.46	93.36

## 4 DISCUSSION

It is well established that structure of bacteria community in phyllosphere is affected by abiotic factor, such as environmental conditions (solar UV radiation, temperature fluctuations, relative humidity, and nutrition availability) and agronomic practices, and biotic factors, such as host plant species and genotypes (Grube et al., 2011; Gu et al., 2010; Legein et al., 2020; Vorholt, 2012). This study explored the influences of tobacco varieties and ecological regions on the composition and diversity of bacterial communities in phyllosphere of harvested tobacco using 16s rRNA gene sequencing. The results revealed that there were significant differences as to the alpha diversity between bacterial samples from different geographic locations. However, the beta diversity and community structure of phyllosphere bacteria were not significantly affected by geographic locations and tobacco genotypes. The obvious effect of geographic distance on bacterial diversity has been widely reported, which was mainly attributed to site‐specific factors such as nutrient availability, temperature fluctuations, relative humidity, and solar radiation (Xiong et al., 2020). In a field trial, Darlison et al. (2019) surveyed the bacterial communities of rocket (*Diplotaxis tenuifolia*) and baby leaf spinach (*Spinacia oleracea*) with four doses of nitrogen fertilizer. It was found that alpha diversity of bacterial communities decreased with the increase in nitrogen fertilizer dose. Importantly, annual variations were found to have the strongest effect on the bacterial community. Chen et al. (2018) observed that long‐term application of chicken manure and sewage sludge altered the composition of bacterial community in phyllosphere, resulting in an obvious decrease as to bacterial alpha diversity. It was also suggested that the excess of soil nutrient levels might induce lower microbial diversity due to the nutrient accumulation (Martirosyan et al., 2016). Moreover, Redford et al. (2010) suggested that tree species had their own distinctive structure composition of bacterial community in phyllosphere, which unchanged even when these trees were planted in different areas in the world. We speculate that the species of host plant and annual variations might pose a more significant impact on the structure of bacterial community in phyllosphere compared with cultivation sites. This may explain why the cultivation sites had no remarkable effect on beta diversity and community structure of phyllosphere in our study.

In addition, genetic background of tobacco might have certain effects on the diversity and structure of bacterial community in phyllosphere, possibly by leaf surface properties or jasmonic acid/γ‐aminobutyric acid signaling pathway (Chen et al., 2020; Vorholt, 2012). Kim et al. (2012) observed that the similarity of phyllosphere bacterial community among these tree species was determined by the host plant phylogeny and more similar communities in closely related host plants. Xiong et al. (2020) discovered that election effect of host plants reduced bacterial diversity. Chen et al. (2021) demonstrated that bacterial communities in the tobacco phyllosphere possessed a high resilience toward environmental changes. These studies might indicate that the same host plant possessed similar structure of bacterial community and the effect of tobacco varieties on the structure of bacterial community would not be remarkable.

The DNA sequencing showed that *Proteobacteria* was the most abundant phylum among all samples, followed by *Cyanobacteria*. *Proteobacteria* is the most common phyllosphere bacteria in numerous crops such as spinach (Wellner et al., 2011), flue‐cured tobacco (Huang et al., 2010), tea (Cernava et al., 2019), and rice (Thapa et al., 2017). *Proteobacteria* is capable of colonizing various niches like rhizosphere and phyllosphere, which could explain their dominant distribution (Xiong et al., 2020). The huge abundance of *Cyanobacteria* in the phyllosphere was not reported in the previous reports (Chen et al., 2021). *Cyanobacteria* is a photosynthetic prokaryote and beneficial to soil fertility and crop production because of its ability to solubilize phosphate, fix atmospheric nitrogen, and generate plant growth regulators (Toribio et al., 2020). The major bacteria at the genus level were *Limnobacter*, *Brevundimonas*, unidentified_*Cyanobacteria*, and *Pseudomonas*; the result was not similar with previous studies. Chopyk et al. (2017) found that bacterial communities were dominated by *Pseudomonas*, *Bacillus*, and *Pantoea* in five commercial tobacco brands, regardless of storage condition. Huang et al. (2010) reported that the main bacterial genera in unaged and aging flue‐cured tobacco were *Bacillus* and *Pseudomonas*. Chen et al. (2021) found that the genera *Sphingomonas* and *Pseudomonas* were the most abundant colonizers in the tobacco leaves, as they can synthesize extracellular polysaccharides and specific pigments to protect them from osmotic stress and UV radiation. Although the effects of sites and varieties on community structures were not significant, there were significant differences between groups in the genus levels. Similarly, Knief et al. (2010) indicated that site‐specific factors showed a stronger effect on the *Methylobacterium* composition than plant species‐specific factors.

Some bacteria have formed specific viability to adapt to the plant phyllosphere. It was found that SO_4_^2‐^ acted as an electron acceptor when NO_3_^‐^ and NO_2_^‐^ were deficient and led to the increase in sulfur‐related bacteria, for example, *Limnobacter* (Kang et al., 2018). *Brevundimonas* showed high survival rates under oligotrophic conditions and was able to degrade dimethoate and quinolone (Song et al., 2019). Therefore, the dominance of genera *Limnobacter* and *Brevundimonas* in this study might be ascribed to nutrient scarcity on the surface of tobacco leaves. *Pseudomonas* can reach more favorable sites by flagellar motility, synthesize the biosurfactant osmoprotectants to keep water on the leaf surface, and apply effectors to make water from the cells leak into the apoplast (Legein et al., 2020). Therefore, its relative abundance remained stable among the groups in the study. *Sphingomonas* withstands the scarcity of nutrients by utilizing a wide range of carbon sources (Vorholt, 2012). Accordingly, *Sphingomonas* showed more abundance in the nutrient scarcity region of GC. The functions of some bacteria on the tobacco curing have been clarified in previous reports also. *Pseudomonas* had a positive effect on the degradation of nicotine and can produce the high contents of lipopolysaccharide in cigarette tobacco and smoke (Chopyk et al., 2017).

In general, the interactions between soil nutrients and microbial communities of soil are well known (Thapa et al., 2017). For example, *Proteobacteria* and *Actinobacteria* participated in the P solubilization for the tobacco plants. Deficiency of P in the soil would limit soil bacterial diversity and abundance (Lisuma et al., 2020). The effect of soil nutrients on plant growth and the interactions between leaf mineral contents and phyllosphere bacterial communities are also well documented (Laforest‐Lapointe & Whitaker, 2019). Moreover, most of the researchers focus on the climatic factors when investigating the effect of geographic locations on the phyllosphere bacterial communities (Bell & Wagstaff, 2014). Importantly, the phyllosphere is an open environment and phyllosphere microorganisms might derive from soil through wind, rain splash, or human action in agronomic measures (Vorholt, 2012). Therefore, this study attempted to establish the possible relations between soil nutrient status and bacterial communities in the plant phyllosphere. Correlations between soil macronutrient status/climatic factors and phyllospheric bacterial population were primarily negative in the study. Analyses of correlations between soil macronutrient status and phyllospheric population can be complex, as investigated in the other reports. Thapa et al. (2017) surveyed that all the leaf macronutrients in the seven rice varieties exhibited positive correlations with respective soil nutrients, demonstrating the close relationship between soil nutrient availability and plant uptake. Besides, significantly positive corrections between soil OM/TP and culturable phyllospheric microbial population were found. Darlison et al. (2019) discovered that nitrogen additions led to a significant increase in TN, nitrate, TP, and TK in rocket leaves. However, the significant correlations between bacterial genus abundance and mineral levels of leaves were predominantly negative.

The effect of soil nutrient status on the bacterial population on the leaves might be indirect and mediated by plants. Soil nutrients have substantial effects on plant morphology, biomass, and physiology, which further exert significant influences on the bacterial communities in the plant phyllosphere (Martirosyan et al., 2016; Tang et al., 2020). For instance, plant morphology during the vegetative phase can provide more and larger leaves for microbial colonization (Darlison et al., 2019). The nutrients and bioactive compounds generated on the leaf surface by photosynthesis would also determine the bacterial communities in the plant leaves. Rocket is reported to synthesize various bioactive compounds such as flavonols and glucosinolates (Bell & Wagstaff, 2014). Moreover, the host plants may select bacterial species which can protect them from pathogens/predators or possess traits to colonize this extreme habitat (Thapa et al., 2017). Xiong et al. (2020) demonstrated that microbial communities of wheat phyllosphere were altered primarily by host species and compartment niche rather than by fertilization practices or sites. Source tracking indicated that the crop microbiome predominantly resulted from soils, enriched gradually, and filtered at different plant compartment niches. Chen et al. (2018) also recommended that organic fertilizer application could influence the phyllosphere microbiome through recruiting the manure‐borne bacteria by air. Finally, factors such as chemical composition variations of leaves and leaf exposure to sun would also result in a high phyllosphere microbiome variability over individual plant of the same species (Chen et al., 2021; Lisuma et al., 2020; Truchado et al., 2017). These factors aggravate the exploration of bacterial community response to environmental factors. Further studies will explore the relations between phyllosphere bacteria of tobacco plants and leaf chemical and mineral compositions and investigate the relations of microorganisms in the soils and leaves.

## 5 CONCLUSIONS

This study investigated the bacterial community changes in the phyllosphere of tobacco by 16s rRNA gene sequencing analysis. Our results showed that alpha diversity of bacterial communities was significantly affected by the geographic locations, while beta diversity and community structure were not remarkably influenced by geographic locations and tobacco varieties. The predominant phyla consisted of *Proteobacteria* and *Cyanobacteria* among all the samples, with these genera *Limnobacter*, *Brevundimonas*, unidentified_*Cyanobacteria*, and *Ralstonia* dominating. Environmental factors had greater influence on the composition and diversity of the phyllosphere bacteria than the genetic backgrounds of tobacco plants. Environmental factors mainly showed negative correlations with relative abundance of the bacterial genera. Understanding the dynamics of bacteria in the tobacco leaves provides opportunities to manipulate plant growth and disease and enhance the quality of tobacco leaves in the aging process.

## CONFLICT OF INTEREST

None declared.

## AUTHOR CONTRIBUTIONS

**Lei Xing:** Conceptualization (lead); Data curation (lead); Formal analysis (lead); Investigation (lead); Methodology (lead); Project administration (lead); Resources (lead); Writing‐original draft (lead). **Jianlei Yang:** Conceptualization (equal); Investigation (equal); Resources (equal). **Yuhong Jia:** Conceptualization (equal); Investigation (equal); Resources (equal). **Xi Hu:** Conceptualization (equal); Investigation (equal); Methodology (equal); Resources (equal). **Yi Liu:** Conceptualization (equal); Investigation (equal); Resources (equal). **Heng Xu:** Conceptualization (equal); Investigation (equal); Resources (equal). **Huaqun Yin:** Conceptualization (equal); Resources (equal); Software (equal). **Juan Li:** Investigation (equal); Resources (equal). **Zhenxie Yi:** Conceptualization (lead); Supervision (lead); Validation (lead); Visualization (lead); Writing‐review & editing (equal).

## Supporting information

Table S1

## Data Availability

DNA raw sequences are available in NCBI with the accession number PRJNA71922.

## References

[ece37861-bib-0002] Bell, L., & Wagstaff, C. (2014). Glucosinolates, myrosinase hydrolysis products, and flavonols found in rocket (*Eruca sativa* and *Diplotaxis tenuifolia*). Journal of Agricultural and Food Chemistry, 62, 4481–4492. 24773270 10.1021/jf501096x

[ece37861-bib-0003] Cernava, T., Chen, X., Krug, L., Li, H., Yang, M., & Berg, G. (2019). The tea leaf microbiome shows specific responses to chemical pesticides and biocontrol applications. Science of the Total Environment, 667, 33–40. 10.1016/j.scitotenv.2019.02.319 30825819

[ece37861-bib-0005] Chen, Q. L., An, X. L., Zheng, B. X., Ma, Y. B., & Su, J. Q. (2018). Long‐term organic fertilization increased antibiotic resistome in phyllosphere of maize. Science of the Total Environment, 645, 1230–1237. 10.1016/j.scitotenv.2018.07.260 30248848

[ece37861-bib-0006] Chen, Q. L., Cai, L., Wang, H. C., Cai, L. T., Goodwin, P., Ma, J., Wang, F., & Li, Z. (2020). Fungal composition and diversity of the tobacco leaf phyllosphere during curing of leaves. Frontiers in Microbiology, 11, 554051–554066. 10.3389/fmicb.2020.554051 33013785 PMC7499341

[ece37861-bib-0007] Chen, X., Wicaksono, W. A., Berg, G., & Cernava, T. (2021). Bacterial communities in the plant phyllosphere harbour distinct responders to a broad‐spectrum pesticide. Science of the Total Environment, 751, 141799–141809. 10.1016/j.scitotenv.2020.141799 32889475

[ece37861-bib-0008] Chopyk, J., Chattopadhyay, S., Kulkarni, P., Smyth, E. M., Hittle, L. E., Paulson, J. N., Pop, M., Buehler, S. S., Clark, P. I., Mongodin, E. F., & Sapkota, A. R. (2017). Temporal variations in cigarette tobacco bacterial community composition and tobacco‐specific nitrosamine content are influenced by brand and storage conditions. Frontiers in Microbiology, 8, 358–372. 10.3389/fmicb.2017.00358 28326071 PMC5339245

[ece37861-bib-0009] Darlison, J., Mogren, L., Rosberg, A. K., Gruden, M., Minet, A., Line, C., Mieli, M., Bengtsson, T., Hakansson, A., Uhlig, E., Becher, P. G., Karlsson, M., & Alsanius, B. W. (2019). Leaf mineral content govern microbial community structure in the phyllosphere of spinach (*Spinacia oleracea*) and rocket (*Diplotaxis tenuifolia*). Science of the Total Environment, 675, 501–512. 10.1016/j.scitotenv.2019.04.254 31030156

[ece37861-bib-0010] Edgar, R. C. (2013). UPARSE: Highly accurate OTU sequences from microbial amplicon reads. Nature Methods, 10, 996–1001. 10.1038/nmeth.2604 23955772

[ece37861-bib-0011] Grube, M., Schmid, F., & Berg, G. (2011). Black fungi and associated bacterial communities in the phyllosphere of grapevine. Fungal Biology, 115, 978–986. 10.1016/j.funbio.2011.04.004 21944210

[ece37861-bib-0012] Gu, L., Bai, Z., Jin, B., Hu, Q., Wang, H., Zhuang, G., & Zhang, H. (2010). Assessing the impact of fungicide enostroburin application on bacterial community in wheat phyllosphere. Journal of Environmental Sciences, 22, 134–141. 10.1016/S1001-0742(09)60084-X 20397397

[ece37861-bib-0013] Huang, J., Yang, J., Duan, Y., Gu, W., Gong, X., Zhe, W., Su, C., & Zhang, K. Q. (2010). Bacterial diversities on unaged and aging flue‐cured tobacco leaves estimated by 16S rRNA sequence analysis. Applied Microbiology and Biotechnology, 88, 553–562. 10.1007/s00253-010-2763-4 20645083

[ece37861-bib-0015] Kang, D., Lin, Q., Xu, D., Hu, Q., Li, Y., Ding, A., Zhang, M., & Zheng, P. (2018). Color characterization of anammox granular sludge: Chromogenic substance, microbial succession and state indication. Science of the Total Environment, 642, 1320–1327. 10.1016/j.scitotenv.2018.06.172 30045512

[ece37861-bib-0016] Kim, M., Singh, D., Lai‐Hoe, A., Go, R., Abdul Rahim, R., Ainuddin, A. N., Chun, J., & Adams, J. M. (2012). Distinctive phyllosphere bacterial communities in tropical trees. Microbial Ecology, 63, 674–681. 10.1007/s00248-011-9953-1 21990015

[ece37861-bib-0017] Knief, C., Ramette, A., Frances, L., Alonso‐Blanco, C., & Vorholt, J. A. (2010). Site and plant species are important determinants of the Methylobacterium community composition in the plant phyllosphere. ISME Journal, 4, 719–728. 10.1038/ismej.2010.9 20164863

[ece37861-bib-0018] Laforest‐Lapointe, I., & Whitaker, B. K. (2019). Decrypting the phyllosphere microbiota: Progress and challenges. American Journal of Botany, 106, 171–173. 10.1002/ajb2.1229 30726571

[ece37861-bib-0019] Legein, M., Smets, W., Vandenheuvel, D., Eilers, T., Muyshondt, B., Prinsen, E., Samson, R., & Lebeer, S. (2020). Modes of action of microbial biocontrol in the phyllosphere. Frontiers in Microbiology, 11, 1619–1637. 10.3389/fmicb.2020.01619 32760378 PMC7372246

[ece37861-bib-0021] Lisuma, J. B., Zuberi, Z., Ndakidemi, P. A., & Mbega, E. R. (2020). Linking rhizosphere bacterial diversity and soil fertility in tobacco plants under different soil types and cropping pattern in Tanzania: A pilot study. Heliyon, 6, 04278–04288. 10.1016/j.heliyon.2020.e04278 PMC7347649 32671244

[ece37861-bib-0022] Liu, H., Brettell, L. E., & Singh, B. (2020). Linking the phyllosphere microbiome to plant health. Trends in Plant Science, 25, 841–844. 10.1016/j.tplants.2020.06.003 32576433

[ece37861-bib-0023] Martirosyan, V., Unc, A., Miller, G., Doniger, T., Wachtel, C., & Steinberger, Y. (2016). Desert perennial shrubs shape the microbial‐community miscellany in laimosphere and phyllosphere space. Microbial Ecology, 72, 659–668. 10.1007/s00248-016-0822-9 27450478

[ece37861-bib-0024] Perazzolli, M., Nesler, A., Giovannini, O., Antonielli, L., Puopolo, G., & Pertot, I. (2020). Ecological impact of a rare sugar on grapevine phyllosphere microbial communities. Microbiological Research, 232, 126387–126397. 10.1016/j.micres.2019.126387 31790975

[ece37861-bib-0025] Redford, A. J., Bowers, R. M., Knight, R., Linhart, Y., & Fierer, N. (2010). The ecology of the phyllosphere: Geographic and phylogenetic variability in the distribution of bacteria on tree leaves. Environmental Microbiology, 12, 2885–2893. 10.1111/j.1462-2920.2010.02258.x 20545741 PMC3156554

[ece37861-bib-0026] Song, M., Wang, Y., Jiang, L., Peng, K., Wei, Z., Zhang, D., Li, Y., Zhang, G., & Luo, C. (2019). The complex interactions between novel DEHP‐metabolising bacteria and the microbes in agricultural soils. Science of the Total Environment, 660, 733–740. 10.1016/j.scitotenv.2019.01.052 30743959

[ece37861-bib-0027] Tang, Z., Chen, L., Chen, Z., Fu, Y., Sun, X., Wang, B., & Xia, T. (2020). Climatic factors determine the yield and quality of Honghe flue‐cured tobacco. Scientific Reports, 10, 19868–19880. 10.1038/s41598-020-76919-0 33199769 PMC7669845

[ece37861-bib-0028] Thapa, S., Prasanna, R., Ranjan, K., Velmourougane, K., & Ramakrishnan, B. (2017). Nutrients and host attributes modulate the abundance and functional traits of phyllosphere microbiome in rice. Microbiological Research, 204, 55–64. 10.1016/j.micres.2017.07.007 28870292

[ece37861-bib-0029] Toribio, A. J., Suarez‐Estrella, F., Jurado, M. M., Lopez, M. J., Lopez‐Gonzalez, J. A., & Moreno, J. (2020). Prospection of cyanobacteria producing bioactive substances and their application as potential phytostimulating agents. Biotechnology Reports, 26, 00449–00457. 10.1016/j.btre.2020.e00449 PMC7184136 32368511

[ece37861-bib-0030] Truchado, P., Gil, M. I., Reboleiro, P., Rodelas, B., & Allende, A. (2017). Impact of solar radiation exposure on phyllosphere bacterial community of red‐pigmented baby leaf lettuce. Food Microbiology, 66, 77–85. 10.1016/j.fm.2017.03.018 28576376

[ece37861-bib-0031] Vorholt, J. A. (2012). Microbial life in the phyllosphere. Nature Reviews Microbiology, 10, 828–840. 10.1038/nrmicro2910 23154261

[ece37861-bib-0032] Wellner, S., Lodders, N., & Kampfer, P. (2011). Diversity and biogeography of selected phyllosphere bacteria with special emphasis on *Methylobacterium* spp. Systematic and Applied Microbiology, 34, 621–630. 10.1016/j.syapm.2011.08.005 22000032

[ece37861-bib-0033] Xiong, C., Zhu, Y. G., Wang, J. T., Singh, B., Han, L. L., Shen, J. P., Li, P. P., Wang, G. B., Wu, C. F., Ge, A. H., Zhang, L. M., & He, J. Z. (2020). Host selection shapes crop microbiome assembly and network complexity. New Phytologist, 229(2), 1091–1104. 10.1111/nph.16890 32852792

[ece37861-bib-0034] Yuan, S., Li, M., Fang, Z., Liu, Y., Shi, W., Pan, B., Wu, K., Shi, J., Shen, B., & Shen, Q. (2016). Biological control of tobacco bacterial wilt using *Trichoderma harzianum* amended bioorganic fertilizer and the arbuscular mycorrhizal fungi *Glomus mosseae* . Biological Control, 92, 164–171. 10.1016/j.biocontrol.2015.10.013

[ece37861-bib-0035] Zhao, M., Wang, B., Li, F., Qiu, L., Li, F., Wang, S., & Cui, J. (2007). Analysis of bacterial communities on aging flue‐cured tobacco leaves by 16S rDNA PCR‐DGGE technology. Applied Microbiology and Biotechnology, 73, 1435–1440. 10.1007/s00253-006-0625-x 17043820

