# Supplementary material for: Effects of ecological environment and host genotype on the phyllosphere bacterial communities of cigar tobacco (Nicotiana tabacum L.)
Source: Ecol Evol. 2021 Jul 21;11(16):10892–903. doi: 10.1002/ece3.7861 (PMC8366869; doi:10.1002/ece3.7861)
Supplement: Supplementary file 1 — Table S1 [file ECE3-11-10892-s001.docx]

**Table S1**

Taxa with significant differences between the samples.

| Samples | Genera | Relative abundance | *p* |
| --- | --- | --- | --- |
| GC.M.2&WZS.M.2 | *Melittangium* | 0.19% & 0.0015% | 0.049 |
|  | *Blastococcus* | 0.075% & 0.012%, | 0.024 |
|  | *Roseomonas* | 0.054% & 0.0058% | 0.015 |
|  | *Craurococcus* | 0.012% & 0.0022% | 0.016 |
| GC.M.3&WZS.M.3 | *Melittangium* | 0.13% & 0.0095% | 0.0042 |
|  | *Altererythrobacter* | 0.043% & 0.0044% | 0.012 |
|  | *Mycobacterium* | 0.045% & 0.012% | 0.049 |
|  | *Luteimonas* | 0.022% & 0.0029% | 0.0061 |
|  | *Adhaeribacter* | 0.039% & 0 | 0.012 |
|  | *Gaiella* | 0.017% & 0.0015% | 0.0018 |
|  | *Peredibacter* | 0.0058% & 0 | 0.015 |
|  | *Amycolatopsis* | 0.011% & 0 | 0.013 |
| WZS.M.2&WZS.M.3 | *Brevundimonas* | 39.05% & 27.53%, | 0.044 |
|  | *Devosia* | 1.51% & 0.93%, | 0.0076 |
|  | *Hirschia* | 0.51% & 0.30%, | 0.000051 |
|  | *Agrococcus* | 0.0044% & 0.0095% | 0.036 |
